# Prevalence, Abundance, and Virulence of Adherent-Invasive *Escherichia coli* in Ulcerative Colitis, Colorectal Cancer, and Coeliac Disease

**DOI:** 10.3389/fimmu.2022.748839

**Published:** 2022-03-10

**Authors:** Mireia López-Siles, Carla Camprubí-Font, Eva M. Gómez del Pulgar, Miriam Sabat Mir, David Busquets, Yolanda Sanz, Margarita Martinez-Medina

**Affiliations:** ^1^ Microbiology of Intestinal Diseases, Biology Department, Universitat de Girona, Girona, Spain; ^2^ Instituto de Agroquímica y Tecnología de Alimentos, Spanish National Research Council (CSIC), Paterna, Spain; ^3^ Department of Gastroenterology, Hospital Santa Caterina, Salt, Spain; ^4^ Department of Gastroenterology, Hospital Universitari Doctor Josep Trueta, Girona, Spain

**Keywords:** adherent-invasive *Escherichia coli*, ulcerative colitis, colorectal cancer, coeliac disease, Crohn’s disease

## Abstract

**Background & Aims:**

Adherent-invasive *E. coli* (AIEC) has largely been implicated in the pathogenesis of Crohn’s disease (CD). *E. coli* strains with similar genetic backgrounds and virulence genes profiles have been associated with other intestinal disorders, such as ulcerative colitis (UC), colorectal cancer (CRC), and coeliac disease (CeD), but the role of AIEC in these diseases remains unexplored. We aimed to assess the distribution, abundance, and pathogenic features of AIEC in UC, CRC, and CeD.

**Methods:**

The AIEC phenotype was investigated in 4,233 *E. coli* isolated from the ileum and colon of 14 UC and 15 CRC patients and in 38 fecal *E. coli* strains obtained from 17 CeD and 10 healthy (H) children. AIEC prevalence and abundance were compared with previous data from CD patients and H controls. Clonality, virulence gene carriage, and phylogenetic origin were determined for the AIEC identified.

**Results:**

In UC, AIEC prevalence was intermediate between CD and H subjects (UC: 35.7%, CD: 55.0%, H: 21.4%), and similar to CD patients with colonic disease (C-CD: 40.0%). In CRC, the prevalence was lower (6.7%) than these groups. In patients with AIEC, the estimated abundance was similar across all intestinal conditions. All AIEC strains isolated from UC and CRC belonged to the B1 phylogroup, except for a strain of the A phylogroup, and the majority (75% of clonally distinct AIEC) harbored the Afa/Dr operon and the *cdt* gene. None of the *E. coli* isolated from the CeD cohort were AIEC. Nonetheless, *E. coli* strains isolated from active CeD patients showed higher invasion indices than those isolated from H and inactive CeD pediatric patients.

**Conclusion:**

We support the hypothesis that AIEC-like strains can be involved not only in CD but also in UC. Further works are needed to study the virulence particularities of these groups of strains and to determine if there is a causative link between AIEC and UC. In contrast, we rule out the possible association of AIEC with CRC. In addition, to further study the *E. coli* strains in CeD for their possible pathogenic role would be of interest.

## Introduction

Adherent-invasive *Escherichia coli* (AIEC) comprises a group of genetically diverse *E. coli* with adhesion and invasion abilities and intramacrophage survival and replication capacity, genetically distinct from known intestinal pathogenic *E. coli* pathotypes and close to extraintestinal pathogenic *E. coli* (ExPEC) (1–4). AIEC has been suggested to be implicated in Crohn’s disease (CD) pathogenesis; first, because its pathogenicity mechanisms have been comprehensively linked to many characteristics of CD physiopathology [see reviews (5–10)]; and second, because several independent studies have revealed a higher prevalence of AIEC in CD patients (1, 2, 11–17).

However, the prevalence and putative implication of AIEC in other inflammatory bowel diseases (IBD), such as ulcerative colitis (UC), are still unclear. Few studies have investigated the frequency of UC patients colonized by AIEC strains, but no consensus on distribution has been reached. Prevalence values between 0 and 40% have been reported (1, 15, 16, 18, 19). Some studies show that this prevalence of AIEC in UC is lower than in CD (1, 16), whereas others sustain that it is similar or even higher (15, 18, 19). Moreover, invasion rates of UC- and CD-*E. coli* were found to be similar (20), and intramacrophage replication indices were even higher in UC-*E. coli* than in CD-*E. coli* (21). Therefore, additional studies are needed to elucidate the possible role of AIEC in UC.

Several research groups have suggested a link between *E. coli* and colorectal cancer (CRC), but there is still no study regarding AIEC and CRC. Swidsinski et al. (22) and Martin et al. (23) showed an increased prevalence of CRC patients with intracellular *E. coli* (ranging from 33 to 80%) in comparison with control subjects (<1 to 9%). Increased levels of internalized *E. coli* were also found in CRC tumors compared to normal tissue (24). Bonnet et al. (24) found a relationship between the colonization of mucosa by *E. coli* and poor prognostic factors for CRC development. Besides, Martin et al. (23) reported that hemagglutinating positive *E. coli* were associated with CRC patients (33% vs. 4% in controls) and that hemagglutination correlated with the adhesion ability of the strains but not with invasion. In contrast, hemagglutination-positive *E. coli* from the study of Prorok-Hamon et al. (25) were adherent to HT29 and Intestine-407 (I-407) epithelial cells and frequently able to invade I-407 cells, all characteristics that resemble AIEC. In contrast, Raisch et al. (26) found that B2 *E. coli* strains showed low levels of adhesion and invasion on I-407 cells. Further studies determining the prevalence of AIEC in CRC are needed to corroborate or refute the hypothesis for a putative role for AIEC in CRC.

Similar to what occurs in CD, dysbiosis with increased *E. coli* abundance has been observed for children with coeliac disease (CeD) (27, 28). Isolates of this species in CeD children more frequently belong to B2 and D phylogroups and carry virulence- genes such as pilus P, hemolysin A, and other genes characteristic of ExPEC (29). No data about the adhesion and invasion abilities of *E. coli* isolated from CeD children has been obtained to date. Given the commonalities amongst the dysbiosis regarding *E. coli* populations between CD and CeD, further studies aiming to identify at identifying the AIEC phenotype of CeD *E. coli* isolates are of interest to better define the disease specificity of AIEC.

The present study aimed to determine the prevalence and abundance of AIEC in patients with intestinal disorders, such as UC, CRC, and CeD, and compare it with previous data from CD and healthy (H) controls. This study represents the first report in which AIEC prevalence is assessed in CRC and CeD patients; both ileal and colonic AIEC prevalence and abundance are evaluated in UC patients and compared, using the same methodological approach, with CD patients to finally decipher the disease specificity of AIEC. Pathogenic properties of AIEC strains identified in this study have also been studied by determining the phylogenetic origin and its virulence gene carriage.

## Materials and Methods

### Patients and Specimens

#### UC and CRC Cohorts

Patients suffering from UC (N = 14) and CRC (N = 15) were recruited between 2005 and 2014 in the Hospital Santa Caterina (Salt, Spain) and the Hospital Doctor Josep Trueta (Girona, Spain). Subjects were not exposed to antibiotics for 2 months before colonoscopy. The UC cohort comprises from newly diagnosed patients to patients with a decade of disease history with several relapse episodes and different disease locations and disease activity status. The CRC cohort comprises mainly newly diagnosed patients and diverse tumor locations (sigmoid colon, descendent colon, cecum or rectum) and disease stages (dysplasia, neoplasia, or metastasis). Clinical data of patients are depicted in **Table 1** and **Supplementary Table 1**.

**Table 1:** Characteristics of adult study subjects.

Variable		H adults (N = 28)	CD (N = 20)	UC (N = 14)	CRC (N = 15)
Age (mean years, range)		44.7 [21–80]	33.5 [15–49]	45.4 [27–73]	74.5 [56–86]
Gender (N, %)*	Male	12 (43%)	7 (35%)	8 (57.1%)	12 (80%)
	Female	14 (50%)	13 (65%)	6 (42.9%)	3 (20%)
Smoker (N, %)*	No	nd	3 (15%)	8 (57.1%)	11 (73.3%)
	Former smoker	nd	0	5 (35.7%)	2 (13.3%)
	Yes	nd	8 (40%)	0	2 (13.3%)
CD location (N, %)*	Ileal (I-CD)	na	9 (45%)	na	na
	Ileocolonic (IC-CD)	na	7 (35%)	na	na
	Colonic (C-CD)	na	3 (15%)	na	na
UC extension (N, %)	Proctitis (E1)	na	na	3 (21.4%)	na
	Left-sided colitis (E2)	na	na	9 (64.3%)	na
	Pancolitis (E3)	na	na	2 (14.3%)	na
CRC lesion location (N, %)*	No active disease	na	na	na	1 (6.7%)
	Sigmoid colon	na	na	na	3 (20%)
	Descendent colon	na	na	na	1 (6.7%)
	Cecum	na	na	na	2 (13.3%)
	Rectum	na	na	na	4 (26.7%)
CRC type of lesion (N, %)*	Neoplasia	na	na	na	8 (63.3%)
	Dysplasia	na	na	na	2 (13.3%)
	Severe dysplasia	na	na	na	1 (6.7%)
	Neoplasia and metastasis	na	na	na	1 (6.7%)
Disease activity (N, %)	Active	na	8 (40%)	14 (100%)	na
	Inactive	na	7 (35%)	0	na
Treatment (IBD) (N, %)**	None	na	5 (25%)	2 (14.3%)	na
	Azathioprine	na	7 (35%)	2 (14.3%)	na
	Aminosalicylates	na	1 (5%)	6 (42.9%)	na
	Steroids	na	0	2 (14.2%)	na
	Thiopurine	na	1 (5%)	0	na
	Anti-TNF agent	na	4 (20%)	2 (14.2%)	na
CRC treatment (N, %)*	None	na	na	na	4 (26.7%)
	Surgery	na	na	na	3 (20%)
	Neoadjuvant	na	na	na	4 (26.7%)
Surgical resection (N, %)*	No	nd	14 (70%)	14 (100%)	7 (46.7%)
	Yes	nd	3 (15%)	0	8 (53.3%)
Sample (N, %)	Ileum	9 (32.1)%	4 (20%)	2 (14.3%)	0
	Colon	11 (39.3%)	9 (45%)	8 (57.1%)	15 (100%)
	Ileum + Colon	8 (28.6%)	7 (35%)	4 (28.6%)	0

H, healthy subjects; CD, Crohn’s disease; UC, ulcerative colitis; CRC, colorectal cancer; IBD, inflammatory bowel disease; na, not applicable; nd, no data available.

*Gender was available for 26/28 H subjects; Smoking habit was available for 11/20 CD, and 13/14 UC; Surgical resection information was available for 17/20 CD subjects; CD-location was available for 19/20 patients; CD-behavior was available for 9/20 CD patients; CD-disease activity was available for 15/20 CD patients, Treatment was available for 18/20 CD; CRC lesion location, type of lesion and treatment was recorded for 11/15, 12/15 and 11/15 patients, respectively.

**Subjects with combined therapy have been counted grouped as follows: Anti-TNF agents included patients with Anti-TNF plus steroids, aminosalicylate and enteral nutrition; Steroid included a patient with steroids and azathioprine and a patient with steroid and aminosalycilate.

Biopsies were taken during routine colonoscopy, with sterile forceps from affected and unaffected areas of the colon and/or the ileum for some patients, and immediately placed in sterile tubes without any buffer, maintained at 4°C and processed for *E. coli* isolation in the following 12 h.

#### CD and H Cohorts

Data from H adults (N = 28) and CD patients (N = 20) obtained in a previous study (2) was used for comparison between intestinal diseases. Clinical data of these groups are included in **Table 1** for comparative purposes. Briefly, *E. coli* were isolated from biopsies and all the subsequent methodological approaches performed were the same for both IBDs (CD and UC) and CRC patients.

#### CeD Cohort

*E. coli* strains obtained from CeD patients come from a previous study (29). Briefly, the strains were isolated from pediatric patients, nine active CeD patients on a normal gluten-containing diet, showing clinical symptoms of the disease, positive coeliac serology markers and signs of severe enteropathy by duodenal biopsy examination (untreated CeD group); eight symptom-free coeliac patients (non-active coeliac), who had been on a gluten-free diet for 1–2 years (treated CeD group); and ten H children without known food intolerance (control group). None of the children included in the study were treated with antibiotics for at least 1 month before sampling. After collection, fecal samples were stored at 4°C and analyzed in less than 12 h for *E. coli* isolation.

### Bacteria Isolation and *E. coli* Identification

Enterobacteriaceae were isolated from fresh biopsies of CRC and UC patients as described before (2). Biopsies were subjected to three mild ultrasound-wash cycles to discard both transient and loosely attached bacteria. Each cycle consisted of 30 s at 50 Hz, followed by washing with 1 ml of 1× PBS (phosphate-buffered saline). A mild osmotic shock was applied by incubation for 5 min in distilled water to release any intracellular bacteria. During this time, eukaryotic cells are disrupted without compromising bacterial cells viability. Biopsies and supernatants were cultured in Tryptone Bile X-Glucuronide Medium (TBX, Oxoid, Cambridge, UK). Up to 96 colonies were collected per sample, either glucuronidase positive or negative, and confirmed using the indole assay. *E. coli* from CeD patients were isolated from the stools of children in Violet Red Bile Dextrose (VRBD) agar and identified as previously described (29).

### Identification of AIEC

Following the work of Darfeuille-Michaud et al. (1), isolates considered AIEC in this study were those that presented the ability to adhere to intestinal epithelial cells with an adhesion index (ADH_I) ≥1, to invade intestinal epithelial cells with an invasion index (INV_I) ≥1%, and to survive and/or replicate within macrophages with a replication index (REPL_I) ≥100%. In addition, this definition also pointed out that these *E. coli* lack virulence genes linked to diarrheagenic *E. coli*. Because some toxins such as *cdt* have been reported to be linked to acute diarrhea (30), in the context of this work positive strains for *cdt* and *cnf* have been referred to as “AIEC-like” throughout the manuscript. The methodology to identify putative AIEC isolates and to confirm the phenotype is depicted in the following sections.

#### Qualitative Invasion Assays in 96-Well Plates

As a total of 4,233 isolates were collected, we first performed a screening of putative invasive isolates by a qualitative invasion assay in a 96-well cell culture plate. I-407 (ATCC CCL-6) was seeded at a density of 1 × 10^5^ cells/well and incubated for 20 h. Before infection, cell monolayers were washed twice with 100 µl of PBS, and 100 µl of EMEM medium supplemented with 10% heat-deactivated FBS was added. Bacteria were inoculated with a 96-deep-well replica plater. Three hours after infection, the medium was replaced with a fresh medium containing 100 µg/ml gentamicin and incubated for 1 h. The cells were then lysed with 100 µl of 100% Triton X-100. Five microliter spots of direct cell lysates were applied to a square LB (Luria Bertani) agar plate. Once grown, the spots were classified in four categories according to density, from 0 to 3, ranging from less to more invasive. All isolates of density 3 were tested quantitatively to confirm its invasiveness, except those biopsies that harbored more than ten isolates of category 3, for which we usually analyzed a maximum of ten. Some isolates of category 2 were also assayed to confirm if they were invasive or not.

#### Quantitative Adhesion and Invasion Assays

Quantitative adhesion and invasion assays were performed in triplicate for 269 isolates as described previously (1). Initially, gentamicin susceptibility was confirmed. Then, I-407 were seeded in 24-well plates at a density of 4 × 10^5^ cells/well, and monolayers with a fresh medium were infected at a multiplicity of infection (MOI) of 10. For adhesion, infected cells were incubated for 3 h at 37°C with 5% CO_2_ and then washed five times with PBS. For invasion assay, extracellular bacteria were killed by incubating 1 h with culture medium supplemented with gentamicin (100 µg/ml). Cells were washed twice with PBS before and after this step. For both plates, the cells were lysed for 5 min with 1% Triton X-100 in deionized water, and the number of colony-forming units was determined by plating on LB agar and incubating at 37°C overnight. Assays were performed at least per triplicate in separate experiments. Results were expressed as the mean number of bacteria per intestinal cell (ADH_I) and as the percentage of intracellular bacteria with respect to the initial inoculum (INV_I).

#### Survival and Replication Within Macrophages

All invasive strains were assayed for their capacity to survive and replicate within macrophages. Briefly, J774A.1 macrophages (ATCC TIB-67) were seeded in two 24-well plates at a density of 2 × 10^5^ cells/well and grown in RPMI 1640 supplemented with 10% heat-inactivated FBS for 24 h. A plate to quantify bacterial uptake and a plate to quantify bacteria survival and replication were infected at a MOI of 100. To ease the contact of bacteria with macrophages, plates were centrifuged for 10 min at 900 rpm and incubated for 10 min at 37°C with 5% CO_2_. Extracellular bacteria were killed by incubating 40 min with a culture medium supplemented with gentamicin (100 µg/ml). Cells were washed three times with PBS before and after this step. The plate used to calculate the survival/replication was additionally incubated for 24 h with 20 µg/ml of gentamicin. Cell lysis and bacterial counting were performed as explained for the adhesion and invasion assays. Assays were performed at least per triplicate in separate experiments. Results (REPL_I) were expressed as the mean percentage of the number of bacteria recovered at 24 h compared to the number of intracellular bacteria 1 h post-infection (uptake).

### Confirmation of *E. coli* Identity by Phenotypic and Molecular Methods

Adherent-invasive isolates identified were confirmed to be *E. coli* by performing additional phenotypic tests, which were: lactose, citrate, and urease tests, swarming phenotype; and by quantitative Polymerase Chain Reaction (qPCR) using primers and conditions described in (31). Ct values >35 were considered no amplification.

### Pulsed-Field Gel Electrophoresis (PFGE)

To determine the clonality of 15 AIEC isolates recovered, we performed PFGE as described elsewhere (32). Agarose plugs containing genomic DNA were digested with *XbaI* for 4 h. Electrophoresis was carried out in a CHEF-DR III System for 19 h following the protocols of CDC PulseNet (32). TIFF images were analyzed using GelComparII software.

### Virulence Gene Detection and Phylogenetic Grouping

To characterize virulence features, *E. coli* strains were plated in LB agar media and incubated at 37°C in an anaerobic chamber Whitley DG250 Anaerobic Workstation (Don Whitley Scientific, Inc., Shipley, UK) for 12 h. A colony-PCR targeting multiple genes (*chuA*, *yjaA*, and *tspE4C2*) was used for the phylogenetic group characterization following the method of Clermont et al. (33). The multiplex PCR was performed as previously described (29). Isolates were assigned to phylogenetic groups as follows: ChuA+ YjaA+, group B2; ChuA+ YjaA−, group D; ChuA− TspE4C2+ group B1; ChuA− TspE4C2− group A.

The *E. coli* strains were also characterized for the presence of virulence genes using two sets of multiplex PCR as described by Nowrouzian et al. (34). The first multiplex PCR detected the presence of the virulence-associated genes *fimA* (type-1 fimbriae), *papC* (P fimbriae), *sfaD/E* (S fimbriae), and *draA* (Dr haemagglutinin). The second multiplex PCR detected the presence of *hlyA* (hemolysin), *neuB* (capsule K1), *kfiC* (capsule K5), and *iutA* (aerobactin).

Also, the virulence genes *cdt* and *cnf* were screened by PCR as previously described (35) with the following PCR program: denaturation 94°C - 5 min, 30 cycles of 1 min–94°C, 1 min–55°C, 1 min–72°C, with a final extension step of 10 min–72°C. *Pks* virulence gene was also detected following the same protocol as for the *cdt* and *cnf* genes but annealing temperature was at 60°C.

The *afa/draBC* virulence gene was detected using a modification of the protocol by Mora et al. (36) using the following PCR program: denaturation 94°C - 5 min, 30 cycles of 1 min–94°C, 1 min–58°C, 1 min–72°C, with a final extension step of 10 min - 72°C.

Virulence genes characteristic of diarrheagenic *E. coli* pathotypes (*stx1*, *stx2*, *eae*, *ipaH*, *pCDV432*, *eltA*, and *est*) were also investigated as previously described (37).

The primers used can be checked in **Supplementary Table 2**. All PCRs reactions consisted of a 0.2 mM mix of each deoxynucleoside triphosphate, 1× Taq DNA polymerase buffer, 0.25 μM of each primer, and 2.5 U of Taq polymerase. PCR products were separated in a 2% agarose gel electrophoresis and visualized by using GelRed^®^ Nucleic Acid Gel Stain (Biotium, cat number #41003).

### Cytopathic Effect Assay

I-407 cells were seeded in 24-well plates at a density of 4 × 10^5^ cells/well. Monolayers with fresh medium (EMEM) not containing antibiotics, with inactivated FBS, were infected at a MOI of 10. Infected cells were incubated for 4 h at 37°C with 5% CO_2_ and monolayers were examined by microscopy to search for cytopathic effects. Then, both cells in the supernatants and cells adhered to the well, were harvested from the plate, and subjected to Gibco™ Trypan Blue staining to quantify the percentage of dead cells.

### Statistics

AIEC prevalence data (frequency of patients colonized by AIEC) was compared between groups of patients with Fisher’s exact test. This test was also used to assess gender distribution among groups, distribution of phylogenetic origin, and virulence gene frequency in AIEC strains between groups of patients. Quantitative data such as the abundance of AIEC (proportion of AIEC with respect to total Enterobacteriaceae) or adhesion, invasion, and replication indices were compared by non-parametric tests (Mann–Whitney U-test for comparison of two groups, Kruskal–Wallis test for more than two groups).

## Results

### UC and CRC Patients

#### Characteristics of the Study Subjects

All groups of participants were gender-matched (p = 0.056) (**Table 1**). CRC patients were significantly older than those in any of the other groups (p <0.001), whereas UC subjects were age-matched only with H subjects (p = 0.334). The UC group included mainly subjects with left-sided colitis, with an average disease history of 4.7 ± 5.0 years of duration, and who had suffered on average 1.8 ± 2.0 relapses. At the moment of sampling, two patients had severe disease, whereas the others featured moderate or mild activity, and none was in remission according to both UCDAI (Ulcerative Colitis Disease Activity Index) and Mayo score (**Supplementary Table 1**). Concerning CRC patients, all but one were diagnosed at the moment of sampling and mainly presented neoplasia (63.3%) at the rectum (26.7%) or sigmoid colon regions (20.0%).

#### Mucosa-Associated Enterobacteriaceae Isolates

First, the proportion of indole positive Enterobacteriaceae isolates was analyzed as an approximation to know the proportion of *E. coli* within the set of all isolated Enterobacteriaceae. All individuals in all groups harbored indole positive strains, whereas indole negative strains were not commonly detected. Although not statistically significant, CRC presented the highest prevalence (40%), and CD was the group with the lowest prevalence of patients with indole negative strains (20%) (p >0.05; **Table 2**). Concerning the proportion represented by these indole negative strains among the isolates recovered per subject, the lowest abundance was also reported in CD patients (2.2–5.9%), whereas H subjects featured the highest load (1.0–77.1%), followed by CRC subjects (0.4–75.0%) (p >0.05; **Table 2**).

**Table 2:** Prevalence and abundance of indole-negative isolates obtained from each group of patients.

	Patients with indole negative isolates (%)	Abundance of indole negative isolates in patient with indole negative strains (%)
H (N = 28)	9 (32.1)	1.0–77.1
CD (N = 20)	4 (20.0)	0.5–5.9
UC (N = 14)	5 (35.7)	3.2–20.7
CRC (N = 15)	6 (40.0)	0.4–75.0

H, healthy subjects; CD, Crohn’s disease; UC, ulcerative colitis; CRC, colorectal cancer.

#### AIEC Prevalence

The presence of AIEC was screened in 4,233 Enterobacteriaceae isolates obtained from biopsy samples from UC (N = 14) and CRC patients (N = 15). Around 418 isolates were classified as density 3 in the qualitative invasion assay, and 269 isolates were selected to be assayed quantitatively. Eighteen isolates (16 in UC samples and 2 in CRC samples) were identified as adherent, invasive, and able to survive and replicate inside macrophages (**Supplementary Table 3**). Sixteen adherent-invasive isolates were confirmed to be *E. coli*, while two (isolated from the UC patient HSC0013) were not; because they were negative in the indole, lactose, citrate, and urease tests, and also qPCR *E. coli* amplification.

**Figure 1:**
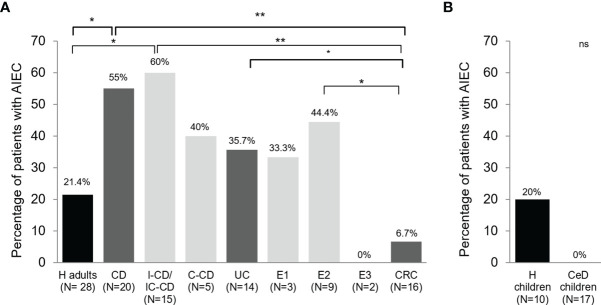
AIEC prevalence in patients with different intestinal diseases and control subjects in biopsy samples **(A)** and feces **(B)**. Prevalence has been calculated as the frequency of patients with at least one AIEC or AIEC-like strain recovered from any of the samples analyzed. Data from Crohn’s disease (CD) patients and healthy adults controls (H) has been extracted from a previous study (2). Bacteria from pediatric patients were previously isolated from fecal samples (29). IC-CD, ileocolonic Crohn’s disease; I-CD, ileal Crohn’s disease; C-CD, colonic Crohn’s disease; UC, ulcerative colitis; E1, proctitis; E2, left-sided colitis; E3, pancolitis; CRC, colorectal cancer; CeD, coeliac disease. ns, not significant; *p ≤ 0.05; **p ≤ 0.01.

In terms of AIEC prevalence, only one CRC patient harbored AIEC-like isolates among the 15 subjects studied (6.7%), while five out of 14 UC patients presented AIEC-like isolates (35.7%). To compare, data obtained in a previous study from H adults (N = 28) and CD patients (N = 20) (2) was included. Differences in AIEC prevalence were observed between intestinal diseases. UC group had higher AIEC prevalence than the H group (21.4%) and lower than CD patients (55.0%); however, no statistically significant differences were found (p >0.05). Besides, AIEC prevalence in UC patients was close to that of the colonic-CD group (C-CD, 40.0%) (**Figure 1A**). AIEC-like isolates were found in UC patients with proctitis (E1; 33.3%) and left-sided colitis (E2; 44.4%), but not in patients with pancolitis (E3). However, only two patients with this disease localization were included in the study. In CRC patients, the AIEC prevalence was very low. In particular, it was significantly lower than in the CD group (p = 0.0035) and the UC group without reaching statistical significance (p = 0.0725), specially the E2 patients (p = 0.0403) (**Figure 1A**). Despite being lower in the latter, no differences in prevalence were found between the H and CRC groups (p >0.3930).

In terms of biopsy location, for those groups that ileal and colonic samples were obtained (H, UC, and CD), no differences in AIEC prevalence were found between regions (**Table 3**) (p >0.05). For the proctitis subtype, a putative difference was suggested since 50% of ileum samples contained AIEC while none of the colonic samples did. However, a low number of samples have been studied (**Table 3**).

**Table 3:** AIEC prevalence (frequency of patients with AIEC and/or AIEC-like strains) and abundance (estimated AIEC and/or AIEC-like isolates/total Enterobacteriaceae) according to biopsy location.

Condition	AIEC Prevalence		AIEC Abundance*	
	Ileum (N)	Colon (N)	Ileum (N)	Colon (N)
**H**	**17.6 (17)**	**15.8 (19)**	**0.203 ± 0.272 (3)**	**0.060 ± 0.043 (3)**
**CD**	**54.5 (11)**	**50.0 (16)**	**0.089 ± 0.180 (6)**	**0.107 ± 0.160 (8)**
I-/IC-CD	55.5 (9)	58.3 (12)	0.104 ± 0.196 (5)	0.117 ± 0.171 (7)
C-CD	50.0 (2)	25.0 (4)	0.011 (1)	0.037 (1)
**UC**	**33.3 (6)**	**30.8 (13)**	**0.045 ± 0.004 (2)**	**0.065 ± 0.095 (5)**
E1	50.0 (2)	0.0 (3)	0.048 (1)	–
E2	50.0 (2)	44.4 (9)	0.042 (1)	0.065 ± 0.095 (5)
E3	0.0 (2)	0.0 (1)	–	–
**CRC**	**–**	**6.7 (15)**	**–**	**0.019 ± 0.012 (2)**

H, healthy subjects; CD, Crohn’s disease; UC, ulcerative colitis; CRC, colorectal cancer; I-CD, ileal Crohn’s disease; IC-CD, ileocolonic Crohn’s disease; C-CD, colonic Crohn’s disease; E1, proctitis; E2, left-sided colitits; E3, pancolitis.

*Abundance (mean ± standard deviation) was calculated only including those patients carrying AIEC and/or AIEC-like strains.

Regarding affectation of the location sampled, similar AIEC prevalence was found between affected and unaffected colon, either for UC (23.1% vs. 25.0% respectively, p >0.05) and CRC (6.7% vs 6.7% respectively, p >0.05) patients. Noticeably, in UC patients, AIEC-like isolates were also found in ileal samples (2/6 biopsies) despite being non-affected.

#### Estimated AIEC Abundance

The estimated abundance of AIEC-like isolates (i.e., ratio of AIEC to total Enterobacteriaceae = [(a/b) × c]/d) was calculated by extrapolating the ratio of confirmed AIEC phenotype (a) to the strains analyzed from category 3 (b) by all strains classified in category 3 (c), and this was divided by the total number of isolated Enterobacteriaceae (d).

**Figure 2:**
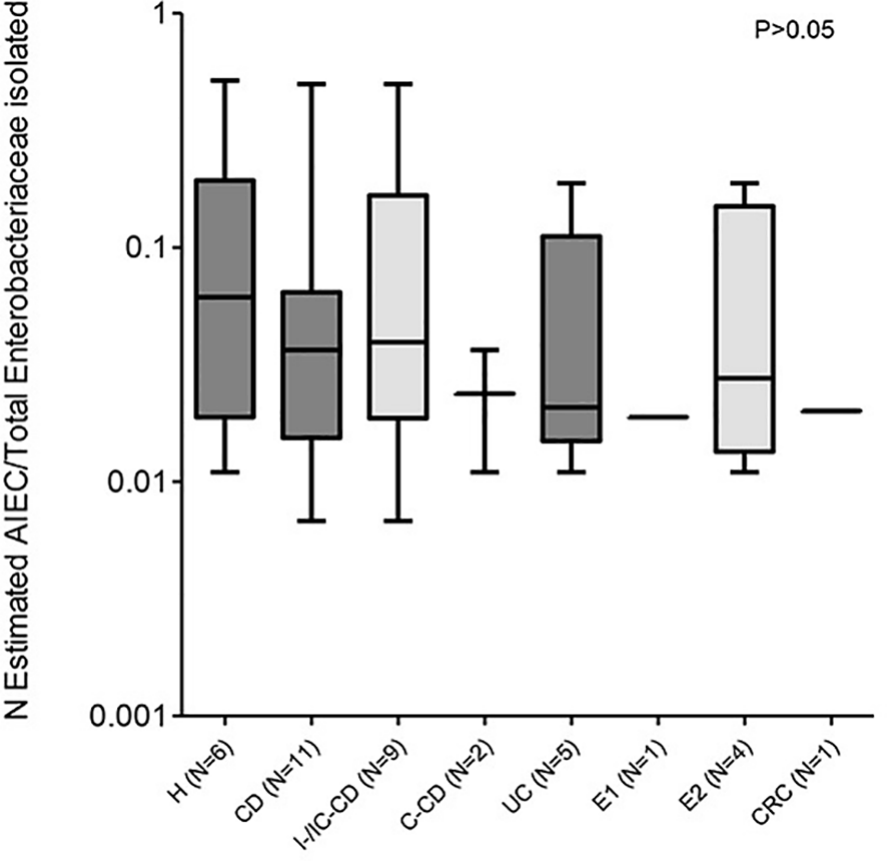
AIEC abundance in patients with different intestinal diseases and control subjects. Abundance has been calculated as the proportion of AIEC and/or AIEC-like isolates with respect to the total Enterobacteriaceae isolates analyzed. Only data from patients with AIEC and/or AIEC-like has been considered. Data from Crohn’s disease (CD) patients and healthy controls (H) has been extracted from a previous study (2). Values indicate the abundance of patients with AIEC strains. For subjects with more than one sample analyzed, the mean abundance of all samples was used for calculations. IC-CD, ileocolonic Crohn’s disease; I-CD, ileal Crohn’s disease; C-CD, colonic Crohn’s disease; UC, ulcerative colitis; E1, proctitis; E2, left-sided colitis; CRC, colorectal cancer.

In patients with isolates with AIEC phenotype, the estimated abundance was similar across all intestinal conditions studied (p >0.05) (**Figure 2**). Despite the limited number of samples, we analyzed the results by location and inflammation status of the tissue because these aspects are relevant to the disease course and/or possible confounding factors. In terms of biopsy location, in UC patients, similar AIEC abundances were reported between ileal (0.0450 ± 0.0037; N = 2) and colonic (0.0651 ± 0.0955; N = 5) biopsies (p >0.05) (**Table 3**). Similarly, AIEC abundances did not differ according to biopsy location for H subjects and CD patients (p >0.05) (**Table 3**). Once comparing between disease conditions, both ileal and colonic biopsies reported similar AIEC abundance (p >0.05). In addition, no differences regarding affected or non-affected colonic zones were reported in UC samples (affected: 0.0236 ± 0.0142 vs non-affected: 0.0862 ± 0.0999; N= 3 and 4, respectively) (p >0.05). For CRC samples, AIEC-like isolates were found only in one affected location (0.0110) and one non-affected location (0.0279). Thus, no statistical analysis on AIEC abundance could be performed.

#### AIEC Characterization

**Figure 3:**
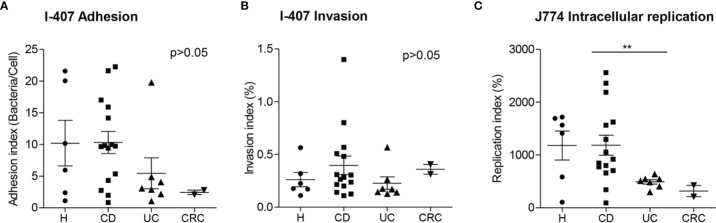
Adhesion **(A)**, invasion **(B)** and replication indices **(C)** of AIEC and AIEC-like clones isolated from patients with different intestinal diseases and control subjects. Data from Crohn’s disease (CD) patients and healthy controls (H) has been extracted from a previous study (2). Only clonally independent strains are represented. A representative isolate has been chosen in cases that more than one isolate corresponded to the same clone. UC, ulcerative colitis; CRC, colorectal cancer. **p ≤ 0.01.

CRC- and UC-AIEC-like isolates were further analyzed to determine their Pulsed-Field Gel Electrophoresis (PFGE) clonality, phylotype, and virulence gene carriage (**Table 4** and **Figure 3**).

**Table 4:** Virulence genes and phylogenetic group of AIEC and AIEC-like strains obtained from ulcerative colitis (UC) and colorectal cancer (CRC) patients.

Disease	Isolate	Patient	Phylogroup	*kfiC*	*iutA*	*hlyA*	*neuB*	*fimA*	*papC*	*sfaD/E*	*afa/draBC*	*draA*	*cdt*	*cnf*	*pks*	ADH_I (bacteria/cell)	INV_I (%)	REPL_I (%)
UC	PL23F02	P107	A	−	−	−	−	−	−	−	−	−	+	−	−	19.8 ± 5.3	0.568 ± 0.006	641.8 ± 319.0
UC	PL40G06	P121	B1	−	+	+	−	+	−	+	−	+	+	+	−	1.1 ± 0.2	0.142 ± 0.064	515.9 ± 335.7
UC	GENAIEC1E01 (•)	HSC003	B1	−	−	−	−	+	−	−	−	+	+	−	−	2.7 ± 1.7	0.201 ± 0.116	191.8 ± 136.4
UC	GENAIEC2A03 (•)	HSC003	B1	−	−	−	−	+	−	−	−	+	+	−	−	7.3 ± 2.5	0.377 ± 0.089	355.4 ± 208.2
UC	GENAIEC3A06 (•)	HSC003	B1	−	−	−	−	+	−	−	−	+	+	−	−	6.1 ± 3.0	0.165 ± 0.081	211.6 ± 80.3
UC	GENAIEC3A08 (•)	HSC003	B1	−	−	−	−	+	−	−	−	+	+	−	−	9.4 ± 3.6	0.150 ± 0.034	465.0 ± 284.4
UC	GENAIEC3A09 (•)	HSC003	B1	−	−	−	−	+	−	−	−	+	+	−	−	4.9 ± 1.6	0.265 ± 0.134	411.0 ± 102.1
UC	GENAIEC13H3	HSC009	B1	−	+	−	−	+	−	+	−	−	−	−	−	2.9 ± 2.4	0.172 ± 0.034	302.3 ± 59.1
UC	GENAIEC43B3 (*)	HT003	B1	+	−	−	−	+	−	−	+	+	+	−	+	4.2 ± 2.2	0.188 ± 0.101	474.6 ± 128.4
UC	GENAIEC43B6 (■)	HT003	B1	+	−	−	−	+	−	−	+	+	−	−	+	6.1 ± 4.0	0.150 ± 0.078	403.1 ± 399.5
UC	GENAIEC43E4 (*)	HT003	B1	+	−	−	−	+	−	−	+	+	+	−	+	4.1 ± 0.8	0.129 ± 0.054	487.6 ± 89.5
UC	GENAIEC43E9 (¤)	HT003	B1	+	−	−	−	+	−	−	+	+	−	−	+	3.1 ± 2.6	0.146 ± 0.026	547.8 ± 157.7
UC	GENAIEC43F5 (■)	HT003	B1	+	−	−	−	+	−	−	+	+	+	−	+	2.2 ± 0.9	0.177 ± 0.072	547.8 ± 157.7
CRC	GENAIEC41B6 (◊)	GENAIEC21	B1	−	−	−	−	+	−	−	−	+	+	−	−	2.1 ± 0.9	0.313 ± 0.146	425.2 ± 278.2
CRC	GENAIEC42B1 (◊)	GENAIEC21	B1	−	−	−	−	+	−	−	−	+	+	−	−	2.8 ± 2.1	0.406 ± 0.472	210.3 ± 133.0

Symbols indicate those strains from a given subject that showed identical XbaI-pulsotype, (see **Supplementary Figure 1**).

ADH_I, adhesion index; INV_I, invasion index ; REPL_I, replication index.

*Virulence genes and phylogenetic group available only for 15/16 adherent-invasive isolates confirmed to be *E. coli.*

A total of nine AIEC-like clones were identified by PFGE (seven from UC and two from CRC patients) (**Supplementary Figure 1**). More than one AIEC-like isolate was obtained in two out of the five UC patients with this bacteria. In one case (HSC003), all the five isolates presented the same pulsotype, while in the other subject (HT003), three different pulsotypes were found among the five AIEC isolates. For the CRC group, the two AIEC clones were isolated from a single patient (GENAIEC13), and they were closely related since only one band of difference was found in the PFGE fingerprint.

All AIEC-like strains were classified in the B1 phylogroup, except for PL23F02, isolated from a UC patient, that was phylogroup A (**Table 4**). The phylogenetic distribution of UC- and CRC-AIEC-like strains differed from CD-AIEC strains (p= 0.003) since 73.3% of CD strains were from the B2 phylogroup and 13.3% from the D phylogroup, two phylogroups that were not represented in UC-AIEC-like strains or in CRC-AIEC-like strains. AIEC strains isolated from H subjects were distributed across A (33.3%), B1 (16.7%), and B2 (50.0%) phylogroups. These showed differences with UC-AIEC-like strains since the most represented phylogroup, in this case, was B1 (85.7%) (p = 0.032).

In terms of virulence genes, AIEC-like strains were negative for all the genes tested of diarrheagenic pathotypes of *E. coli*. As expected, the *fimA* gene was present in almost all the strains (85.7% of UC-AIEC-like strains and 100% of CRC-AIEC-like strains). In turn, *draA* and *cdt* genes were highly prevalent (five out of seven clonally distinct AIEC strains isolated from UC and the two strains isolated from CRC patients). *KfiC*, *pks*, and *afa/draBC* genes were found in three AIEC-like clones of one UC patient, while *iutA*, *sfaD/E*, *hlyA*, and *cnf* genes were found in only one or two UC isolates and were absent in CRC strains. *NeuB* and *papC* were absent in all strains (0% in both UC and CRC). No differences in virulence gene prevalence were found between UC- and CRC-AIEC-like isolates (p >0.05). Similar virulence gene frequencies were also found between UC-AIEC-like and H-AIEC strains and between UC-AIEC-like and CD-AIEC strains, except *papC* which was present in 66.7% of CD-AIEC and absent in UC-AIEC (p = 0.031).

In the cell–bacterium-interaction test none of the strains induced cytopathic effects on I-407 cells, except for the strain PL23F02. This strain induced obvious changes in cell morphology, in particular cell rounding and detachment. Microscopic observations were confirmed by trypan blue staining, since 31.5% of cells were dead in PL23F02-infected monolayers, whereas for the other strains cell death ranged between 1 and 9%.

We compared the adhesion, invasion, and replication indices of UC- and CRC-AIEC-like isolates with the indices of CD and H AIEC strains previously isolated (2) (**Figure 3**). AIEC/AIEC-like strains isolated from different intestinal conditions showed similar values for adhesion and invasion capacities (ADH_I: H = 10.2 ± 8.8, CD = 10.3 ± 6.7, UC = 5.4 ± 6.4, CRC = 2.45 ± 0.5; INV_I: H = 0.262 ± 0.164, CD = 0.397 ± 0.336, UC = 0.228 ± 0.156, CRC = 0.359 ± 0.066; p >0.05). Noticeably, lower replication values were observed for UC-AIEC-like isolates (493 ± 109%) in comparison to CD-AIEC isolates (1,187 ± 735%) (p = 0.003), whereas no differences were observed between AIEC-like isolates from CRC (318 ± 152%) and H (1,180 ± 673%) groups.

### CeD Children

#### Description of the Study Subjects

This cohort included three groups of children from a previous study (29). The mean age of the untreated CeD group was 3.86 years (range 1.0–8.86 years), treated CeD children’s mean age was 6.2 years (range 1.0–12.0 years), and the control group included children with a mean age of 3.51 years (range 0.1–7.75 years).

#### AIEC Prevalence

AIEC screening was also performed in H children (N = 10) and CeD children (N = 17). A total of 38 *E. coli* isolates were assessed for AIEC phenotype (12 for H controls, 14 for active CeD disease, and 12 for inactive CeD disease patients). Ten out of 38 *E. coli* isolates were adherent (three from H controls, four from active CeD, and three from inactive CeD), but only two were invasive (from H controls) (**Supplementary Table 4**). In this case, no AIEC were reported for CeD children, either from active or inactive patients.

Intriguingly, no *E. coli* strains isolated from the 17 CeD children were AIEC, whereas two strains isolated from H children were classified as AIEC (**Figure 1B**). This subset of *E. coli* strains was isolated from fecal samples of pediatric patients. Thus the prevalence cannot be compared with the other intestinal diseases in this study and the previous one in which CD and H adult subjects were analyzed (2). Nevertheless, it was interesting to find out a similar AIEC prevalence between H infants (20.0%) and previous data obtained with H adults (2) (21.4%).

#### Adhesion and Invasion Levels of the Strains

**Figure 4:**
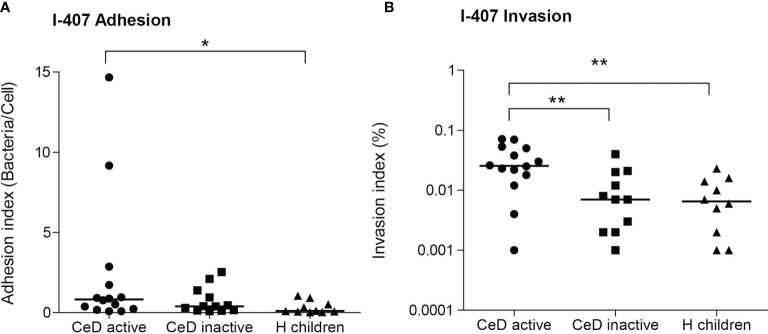
Adhesion **(A)** and invasion **(B)** indices of *E*. *coli* isolates from patients with active and inactive celiac disease (CeD) and healthy (H) children (median indicated). Only non-AIEC strains included (N = 14 active CeD; N = 12 inactive CeD; N = 10 H children). *p ≤ 0.05; **p ≤ 0.01.

The adhesion and invasion indices were assessed for all isolates (N = 38) (**Supplementary Table 4**). Interestingly, non-AIEC strains isolated from active CeD patients (0.0315 ± 0.0223%; 2.4 ± 4.2 bacteria/cell) showed higher invasion and adhesion indices than those isolated from H children (0.00883 ± 0.0074%; 0.33 ± 0.38 bacteria/cell) (p = 0.0049; p = 0.028) (**Figure 4**). Moreover, non-AIEC strains isolated from active CeD patients also presented higher invasion index than those from inactive CeD patients (0.0102 ± 0.0119%) (p = 0.0059) but similar adhesion levels (0.75 ± 0.82 bacteria/cell) (p >0.05). Both AIEC strains isolated from H children presented high invasion indices (CAJ 1 strain: 4.80 ± 0.95 and SAF 3 strain: 1.15 ± 1.02) and moderate adhesion indices (CAJ 1 strain: 3.63 ± 1.69 and SAF 3 strain: 1.27 ± 1.04).

## Discussion

AIEC has been largely linked with CD. However, the association of AIEC with intestinal disorders, such as UC, CRC, and CeD remains unclear or unexplored. In this work, we have investigated for the first time the prevalence of AIEC in CRC and CeD, and we provide new data about the prevalence of AIEC in UC. By massive *E. coli* phenotyping, it is shown for the first time the abundance of AIEC within the *E. coli* population in UC (ileum and colon) and CRC patients (colon). Finally, the new AIEC clones identified here have been characterized by phylo-virotyping.

### *E. coli*/AIEC in Ulcerative Colitis

Although *E. coli* abundance has been related to disease status in UC patients (38, 39) and adherent *E. coli* has been detected in the colon of UC patients (40–43), the AIEC population needs more investigation in larger UC cohorts. In the present study, higher AIEC prevalence in UC patients (35.7%) was reported in comparison with previous studies (0.0–10.0%) (1, 15, 16) but was similar to recent studies conducted in Asia (37.5%) (18) and Australia (40.0%) (19). These studies showed controversial results regarding AIEC prevalence in UC in comparison with CD, since some reported lower prevalence in UC than in CD (1, 16), but others reported similar (18, 44) or even higher prevalence in UC (15, 19). These differences might be due to the size of the cohort studied or the different methodologies applied. We used the same methodology to assess AIEC prevalence and abundance in UC as we did in the previous study for CD and H (2). Therefore, the results are comparable between cohorts. In UC patients, we found an AIEC prevalence lower in comparison with CD patients but higher than H, and no statistically significant differences were found between UC and these two groups of subjects. The prevalence in patients with proctitis (33.3%) and left-sided colitis (44.4%) was similar to the prevalence in CD patients with colonic disease (40.0%) but lower than CD patients with ileal involvement (60.0%). No conclusions can be extracted from patients with pancolitis since only two patients had this condition. Our results are in agreement with a recently published meta-analysis concluding that the AIEC prevalence is higher in UC patients compared to controls (despite not reaching statistical significance), being AIEC a pathobiont that could be involved in both CD and UC pathogenesis (45). Interestingly, 50% of UC patients with proctitis and left-sided colitis had AIEC in the ileum, whereas 0 and 44% of these patients had AIEC in the colon, respectively. AIEC probably colonize more easily the ileum without causing inflammation, and they can later colonize a compromised inflamed colon, perhaps contributing to the disease.

The massive characterization of *E. coli* isolates obtained from UC patients regarding their invasion ability allowed us to estimate the abundance of AIEC strains with respect to the total Enterobacteriaceae population. This approach revealed that the abundance of AIEC is quite similar between UC, CD, or H subjects (**Figure 2**), which suggests that once an individual is colonized by AIEC, this bacterium occupies the niche similarly in all intestinal conditions. Generally, AIEC represents less than 10% of Enterobacteriaceae, except for some individuals (AIEC abundance reached 27 and 50% of Enterobacteriaceae in two I-CD patients, respectively, the 52% in one H subject, and the 19% in a UC patient with mild left-sided colitis).

Regarding the phenotypic characteristics of strains, studies that analyzed the invasion rates of *E. coli* isolates from UC determined that (I) these had lower Caco-2 invasion values (17), (II) these showed similar I-407 (20) and Caco-2 (46) invasion values or (III) these had higher invasion in Hep-2 cells than those *E. coli* isolated from CD patients (18). Although only AIEC strains were quantitatively assessed in the present study, similar UC-AIEC and CD-AIEC I-407 adhesion and invasion values were obtained. UC-AIEC strains had a lower ability to replicate inside J774 macrophages than CD-AIEC. This result is in contrast with previous observations (18, 21). Sepehri et al. (21) reported higher intramacrophage replication in RAW264.7 of UC-isolated *E. coli* in comparison with *E. coli* isolated from control subjects and a moderate higher replication in comparison to CD-*E. coli*. Lee et al. (18) reported similar intracellular survival of CD- and UC-*E. coli* in THP-1. Further studies and meta-analysis are needed to confirm which is the degree of virulence of UC-AIEC on different cellular models.

A meta-analysis that reviews the association of *E. coli* phylogenetic groups with IBD subtypes showed that the B2 phylogroup is frequently associated with IBD, particularly with UC (47). Of note, the AIEC isolated from UC in this study corresponded only to the A or B1 phylogenetic groups. This was an intriguing result considering that AIEC strains isolated from CD patients of the same geographical region are mostly represented by the B2 phylogroup (2).

Although no previous results have compared specifically the virulence gene carriage of AIEC from UC patients with that of AIEC from other disorders, previous reports have shown that UC-*E. coli* have particular virulence genes in comparison with CD- *E. coli* (21, 42, 46, 48, 49), while others showed similar profiles (18, 20, 50, 51). Virulence gene characterization of the strains included in this study indicated that most UC-AIEC strains harbored Afa/Dr adhesins. In comparison with other AIEC strains, it represents a remarkable difference since only 14% of CD-AIEC had genes encoding for the Afa/Dr family adhesins (*afa/draBC* gene tested) (52). Afa/Dr adhesins are commonly present in Diffusely Adhering *E. coli* (DAEC), a pathotype involved in urinary tract infections, pregnancy complications, and acute diarrhea (53). In particular, Dr fimbriae can adhere to several human carcinoembryonic antigen cell adhesion molecules (hCEACAMs), such as CEACAM5 which has been reported to be upregulated in UC patients (54). Therefore, in comparison with AIEC strains present in CD patients, which mostly adhere to human enterocytes *via* the interaction between type 1 fimbriae FimH adhesin and the CEACAM6 receptor (55), this study reveals that AIEC strains present in UC patients may use Dr fimbriae to adhere colonic enterocytes *via* the interaction with human receptors increased in UC such as CEACAM5. Further studies investigating the commonalities and differences between DAEC and AIEC are needed, since DAEC can also invade intestinal epithelial cells *in vitro* (56). However, the ability to survive and replicate in macrophages was used as the defining phenotypic feature to identify AIEC and differentiate them from DAEC strains (57). Another virulence gene that was frequent in strains from UC patients was the *cdt* gene which encodes for toxins with DNase activity (30). None of the CD-AIEC from our previous work harbored this gene (2), but it was present in *E. coli* strains from CRC patients (26). The presence of this gene and the *cnf* gene in the strains excludes them from being classified as AIEC, so we have referred to them as AIEC-like in the manuscript. CDT is a bacterial genotoxin present in gram-negative bacteria that is capable of modulating the eukaryotic cell cycle by pausing the G2/M transition. As a DNase, CDT damages the host DNA activating the DNA damage response. In most cases, the repair system fails to resolve the situation, which leads to cell death or senescence, but a small portion of cells outpace cell cycle arrest and continue to proliferate accumulating DNA lesions, being more likely to develop tumors (58). The high prevalence of this gene in UC-AIEC-like strains is of great relevance considering that UC patients have an increased risk for CRC (59). CDT-producing AIEC colonizing the UC intestine may be responsible of inflammatory response activation and epithelial barrier disruption, but also it could represent a risk for cancer development. Other genotoxins, the cytotoxic necrotizing factor (*cnf*) and the colibactin (*pks*), were also detected in the UC-AIEC strains, yet less frequently. It remains to be determined the prevalence of *cdt*, *cnf* and *pks* in non-AIEC-like *E. coli*, which would be interesting in future studies to assess if this is a commonality in *E. coli* inhabiting the gut of UC patients.

The high prevalence of genotoxins in UC-AIEC-like strains leads us to study the cytopathic effects of these bacteria on intestinal epithelial cells. Only the PL23F02 strain, which belongs to A phylogroup, induced cell death at 4 h post-infection. Of all genes tested, this strain only had the *cdt* gene. CDT-induced cytopathic effects can only be detected in cell–lysate-interaction tests, and are characterized by enlarged nuclei and cell distension (60). Therefore, we suspect that this strain harbors other virulence factors, different from CDT, and is responsible for such cytotoxic effects. Similarly, *E. coli* from the A phylogroup, devoid of known cyclomodulin-encoding genes, were found to induce DNA damage *in vitro* (60).

In summary, AIEC-UC strains identified here had a different genetic profile in comparison with AIEC-CD, being mostly B1 strains with Afa/Dr adhesins and cyclomodulins, characteristics not found in the AIEC-CD investigated previously (2). We hypothesize that AIEC can show a myriad of faces and their virulence particularities may determine the disease type. Nonetheless, we think that a standardized protocol for AIEC identification is needed, as well as further studies comparing the genetic and phenotypic features (e.g., adherence phenotype, invasion abilities, or cytopathic effects caused on host cells, etc.) between close *E. coli* pathotypes, such as AIEC and DAEC, and genotoxin-producing strains.

### *E. coli*/AIEC in Colorectal Cancer

For a long time, intestinal *E. coli* populations have been suspected of playing a role in CRC. Mucosa-associated and intracellular *E. coli* have been more frequently identified in colonic tissue from patients with adenocarcinomas than in controls (22, 23), and within colon cancer more abundance has been found in tumors than in the mucosa (24). Moreover, high levels of mucosa-associated and internalized *E. coli* have been correlated with poor colorectal carcinoma prognostic factors and a higher proliferative index of epithelial cells (24). These strains are frequently cyclomodulin-positive (25, 60, 61) and have pro-carcinogenic activities in murine models (24, 62–65) and human organoids (66).

Few works have addressed the adhesion, invasion, and intracellular replication abilities of CRC-associated *E. coli*. Buc et al. found no differences in adhesion abilities of *E. coli* strains isolated from CRC or diverticulosis patients (60). On the other hand, Sobieszczańska et al. showed that *E. coli* isolated from a pediatric cohort with polyposis had the highest invasion efficiency on I-407 cells compared with *E. coli* strains isolated from IBD children (20). Raisch et al. (26) determined the adhesion and invasion abilities of B2 *E. coli* isolated from CRC and diverticulosis patients, and they found that both adhesion (below 4) and invasion (below 0.5%) levels were much lower for CRC-*E. coli* in comparison with the AIEC reference strain LF82 (adhesion: 53.23 ± 6.63, invasion: around 8%). However, it is unclear whether the strains could be considered AIEC or not following the criteria described in (1), since strains with adhesion levels higher than 1 and invasion levels higher than 0.1% were found, but macrophage intracellular replication was not studied. Darfeuille-Michaud et al. (1) found a low prevalence of AIEC in ileal specimens of right colonic cancer patients (6.2%). Only one out of 16 patients harbored AIEC (one strain with an adhesion level of 16 and an invasion level of 0.2% in I-407 cells and an intramacrophage replication level of 833 in J774 cells). The AIEC prevalence observed in our study was similar to these results since we found that AIEC was present only in 6.7% of CRC patients, which was much lower than AIEC prevalence in UC (35.7%) and CD (60.0%) patients, and similar to H controls. In comparison with AIEC strains isolated from CD and H subjects, CRC-AIEC-like strains of the present study showed lower adhesion values to intestinal epithelial cells but similar invasion abilities, and similar intramacrophage replication indices (**Figure 3**). Thus, our results suggest no particular relationship between AIEC and CRC.

Prorok-Hamon et al. reported that *E. coli* from CRC patients frequently present *afaC* and *pks* genes, and the presence of these virulence genes correlated with higher adhesion and invasion capacities (25). In the present study, only AIEC-like strains were characterized in terms of virulence gene carriage, and we found that none of the two CRC-AIEC-like strains harbored these genes. Nonetheless, it does not rule out the possibility that other non-AIEC strains of the CRC gut frequently harbor these genes. The CRC-AIEC-like strains isolated here harbored *draA* and *cdt* genes. As explained in the previous section, CDT is a genotoxin that may be responsible for tumor induction. Nonetheless, although B2 cyclomodulin producing *E. coli* has been largely incriminated in cancer induction, further evidence is needed to prove this hypothesis or demonstrate whether the flourishment of these strains in the mucosa of CRC patients is an effect of the disease (67). To establish the prevalence of *cdt*, *cnf*, and *pks* in non-AIEC-like *E. coli* from CRC patients will be of interest to know if this is a widely distributed feature in the strains colonizing the gut affected by colorectal cancer.

### *E. coli*/AIEC in Coeliac Disease

Dysbiosis characterized by increased *E. coli* abundance has been observed in untreated CeD children (27, 28). Also, an increased virulence-gene carriage has been reported in *E. coli* isolates from treated and untreated pediatric patients compared to healthy controls (29). However, no previous studies assessing adhesion, invasion, and intracellular replication abilities of *E. coli* present in the gut of CeD patients have been reported. We have phenotypically characterized the *E. coli* strains isolated in a previous study from the feces of children with active CeD, symptom-free CeD patients, and H children (29).

AIEC prevalence in H children was very similar to that previously observed in H adults [20.0% vs. 21.4% (2)], which was noticeable considering the differences between the cohorts compared. For example, *E. coli* from H children were isolated from fecal samples, whereas those of CD patients were obtained from biopsies, and the number of strains screened was much higher for the latter. Nonetheless, this evidences that AIEC might also be present in the gut during childhood and possibly infancy. Surprisingly, none of the CeD patients had AIEC. Thus, we rule out the hypothesis that AIEC could be associated with CeD. However, we are aware that a reduced number of *E. coli* strains have been studied, and these strains have been isolated from feces instead of mucosal samples. Therefore, this hypothesis should be further confirmed. In turn, the fact that the 20% of strains from H children were AIEC in contrast with the 0% from CeD suggests that other types of bacteria are favored in the CeD intestine.

Although no AIEC strains were identified in CeD patients, interestingly, we found that the invasion abilities of the strains of *E. coli* isolated from CeD patients with active disease presented higher invasion ability than those isolated from symptom-free CeD and non-AIEC isolated from H patients (**Figure 4**). On average, CeD active-*E. coli* showed invasion indices almost four times higher than strains of the other two groups. We suggest that the intestinal inflammatory state of untreated CeD patients may generate a more permissive environment that facilitates the colonization of more virulent *E. coli* strains, with a phenotype with increased invasion and/or intracellular survival capacity on intestinal epithelial cells. In turn, these virulent *E. coli* strains can contribute to the inflammatory state creating a vicious circle. In fact, the contribution of an *E. coli* strain isolated from CeD children triggering the gluten-induced immunopathology in mice has been proven (68). Nonetheless, further studies should be warranted to understand how the persistence of an inflammatory state could eventually displace non-virulent *E. coli* strains in favor of more virulent strains or exert a selective pressure driving the evolution of existing *E. coli* strains towards more invasive phenotypes. The study of AIEC prevalence in CeD adult patients with a long history of the disease would also be highly interesting.

## Conclusion

AIEC has for a long time been incriminated to be implicated in ileal CD. Here we show that AIEC-like strains are considerably prevalent and abundant also in other IBD phenotypes, such as UC or colonic CD. Notwithstanding, our study supports that strains from UC and those from CD have different genetic features. In turn, we rule out the possible association of AIEC with CRC, and despite no association being found with CeD, we consider it of interest to further study the evolution of intestinal CeD-*E. coli* populations towards an AIEC phenotype.

## Data Availability Statement

The raw data supporting the conclusions of this article will be made available by the authors, without undue reservation.

## Ethics Statement

The studies involving human participants were reviewed and approved by the CEIC-Institut d’Assistència Sanitària, CEIC-Hospital Universitari de Girona Doctor Josep Trueta, and the CEIC-Hospital General Universitario La Fe of Valencia. Written informed consent to participate in this study was provided by the participant’s legal guardian/next of kin.

## Author Contributions

MLS: Processed biopsies, performed microbiological analyses, revised the manuscript. CCF: Analyzed data, drafted the manuscript, and revised the final version. EGdP: Performed molecular analyses (virulence gene detection and phylotyping). MSM and DB: Selected patients, obtained samples, and reviewed the final version of the manuscript. YS: Provided coeliac disease strains, supervised the study, and revised the manuscript. MMM: Conceived and supervised the study, performed microbiological analyses, assessed clonality of isolates, drafted the manuscript, and revised the final version. All authors listed have made a substantial, direct, and intellectual contribution to the work and approved it for publication.

## Funding

This work was funded by the Spanish Ministry of Education and Science through projects SAF2010-15896, SAF2013-43284-P, and SAF2107-82261-P (MINECO/AEI/FEDER/UE) and the grant AGL2017-88801-P from the Spanish Ministry of Science and Innovation (MICINN, Spain). MLS is a Serra Húnter Fellow.

## Conflict of Interest

The authors declare that the research was conducted in the absence of any commercial or financial relationships that could be construed as a potential conflict of interest.

## Publisher’s Note

All claims expressed in this article are solely those of the authors and do not necessarily represent those of their affiliated organizations, or those of the publisher, the editors and the reviewers. Any product that may be evaluated in this article, or claim that may be made by its manufacturer, is not guaranteed or endorsed by the publisher.
