# Supplementary material for: Prevalence, Abundance, and Virulence of Adherent-Invasive Escherichia coli in Ulcerative Colitis, Colorectal Cancer, and Coeliac Disease
Source: Front Immunol. 2022 Mar 10;13:748839. doi: 10.3389/fimmu.2022.748839 (PMC8960851; doi:10.3389/fimmu.2022.748839)
Supplement: Supplementary file 1 [file DataSheet_1.docx]

Supplementary Material

Prevalence, abundance and virulence of adherent-invasive Escherichia coli in ulcerative colitis, colorectal cancer and coeliac disease

Mireia López-Siles, Carla Camprubí-Font, Eva M. Gómez del Pulgar, Miriam Sabat Mir, David Busquets, Yolanda Sanz, Margarita Martinez-Medina.

# Supplementary Tables

**Supplementary Table 1.** Clinical data of ulcerative colitis (UC) and colorectal cancer (CRC) patients included in the study.

| **Patient ID** | **Age**  **(years)** | **Gender** | **Time since the diagnose (years)** | **Num. of relapses** | **Localization of lesions^¥^** | **UCDAI** | **Mayo subscore** | **Type of lesions (TNM classification)** | **Surgical resection** | **Medication** | **Smoking** |
| --- | --- | --- | --- | --- | --- | --- | --- | --- | --- | --- | --- |
| **UC patients** | |  |  |  |  |  |  |  |  |  |  |
| 107 | 27 | Female | 0 | 0 | E2 | 10 | 2 | na | No | Infliximab | No |
| HSC010 | 49 | Male | 0 | 0 | E1 | 3 | 2 | na | No | No | No |
| HSC011 | 43 | Male | 0 | 0 | E2 | 5 | 1 | na | No | No | Former smoker |
| HT003 | 36 | Male | 0 | 0 | E2 | 6 | 1 | na | No | Aminosalicylate | No |
| HSC009 | 52 | Male | 0 | 0 | E1 | 6 | 2 | na | No | No | Former smoker |
| HSC015* | 53 | Male | 1 | 1 | E1 | 8 | 1 | na | No | Corticosteroid + Aminosalicylate | Former smoker |
| HSC013 | 45 | Female | 2 | 2 | E2 | 3 | 1 | na | No | Aminosalicylate | No |
| HSC004 | 45 | Male | 5 | 5 | E2 | 10 | 3 | na | No | Infliximab + Corticosteroid + Aminosalicylate + Enteral nutrition | Former smoker |
| 121 | 50 | Female | 5 | nd | E2 | nd | 2 | na | No | Aminosalicylate | No |
| HSC014 | 41 | Female | 7 | 1 | E2 | 5 | 1 | na | No | Azathioprine | No |
| HSC003 | 73 | Male | 10 | 4 | E2 | 9 | 2 | na | No | Aminosalicylate | Former smoker |
| 76 | 37 | Female | 11 | 3 | E2 | 7 | 2 | na | No | Corticosteroid + Azathioprine | No |
| 157 | 56 | Male | 12 | 5 | E3 | 4 | 1 | na | No | Azathioprine | Former smoker |
| HT002 | 41 | Female | 13 | nd | E1 | 7 | 2 | na | No | Aminosalicylate | No |
| 131 | 41 | Male | nd | nd | E3 | nd | 3 | na | No | Aminosalicylate | nd |
| **CRC patients** | |  |  |  |  |  |  |  |  |  |  |
| HSC006 | 86 | Female | 0 | na | Sigmoid colon | na | na | Neoplasia (T3N1) | Yes | No | No |
| HSC007 | 67 | Male | 0 | na | Descendent colon | na | na | Neoplasia | Yes | No | No |
| HSC012 | 86 | Female | 0 | na | Cecum | na | na | Neoplasia | No | No | No |
| HSC016 | 79 | Male | 0 | na | Rectum | na | na | Severe dysplasia (PtisN0) | No | Surgery | No |
| HSC017 | 78 | Male | 0 | na | Rectum | na | na | Neoplasia | No | Neoadjuvant therapy | No |
| HSC018 | 79 | Male | 0 | na | Sigmoid colon | na | na | Neoplasia (PT1N0) | No | Surgery | No |
| HSC019 | 63 | Male | 0 | na | rectum | na | na | Neoplasia (PT4N1b) | No | Surgery | No |
| HSC020 | 65 | Male | 0 | na | Sigmoid colon | na | na | Neoplasia (PT3N1b) | No | No | No |
| HSC021 | 78 | Male | 0 | na | Rectum | na | na | Neoplasia and metastasis (PT3N0M1) | No | Neoadjuvant therapy | No |
| HSC023 | 80 | Female | 0 | na | Cecum | na | na | Neoplasia (PT3N1M0) | Yes | Neoadjuvant therapy | No |
| HT001 | 75 | Male | 0 | na | Colon | na | na | Dysplasia | Yes | Surgery | Former smoker |
| HT004 | 74 | Male | 0 | na | Sigmoid colon | na | na | Dysplasia | Yes | Surgery | No |
| HT005 | 77 | Male | 0 | na | Rectum | na | na | nd | Yes | Neoadjuvant therapy | Yes |
| HT006 | 56 | Male | 0 | na | Rectum | na | na | nd | Yes | Surgery | Yes |
| 74 | 75 | Male | 2 | na | No active disease | na | na | na | Yes | nd | Former smoker |
| ¥ For UC patients, Montreal classification: E1: proctitis, E2: left-sided colitis, E3: pancolitis | | | | | | | |  |  |  |  |
| * HSC015 is patient HSC009 one year after the first colonoscopy | | | | | |  |  |  |  |  |  |
| nd: no data; na: not applicable | | | |  |  |  |  |  |  |  |  |

**Supplementary Table 2.** Oligo sequences used for multiplex PCRs

| **Primer** | **Sequence (5' to 3')** | **PCR product base pairs (bp)** | **Reference** |
| --- | --- | --- | --- |
| chuA_Fw | GACGAACCAACGGTCAGGAT | 279 | [[1](#_ENREF_1)] |
| chuA_Rv | TGCCGCCAGTACCAAAGACA |  |  |
| YjaA_Fw | TGAAGTGTCAGGAGACGCTG | 211 |  |
| YjaA_Rv | ATGGAGAATGCGTTCCTCAAC |  |  |
| TspE4C2_Fw | GAGTAATGTCGGGGCATTCA | 152 |  |
| TspE4C2_Rv | CGCGCCAACAAAGTATTACG |  |  |
| papC_Fw | GACGGCTGTACTGCAGGGTGTGGCG | 328 | [[2](#_ENREF_2)] |
| papC_Rv | ATATCCTTTCTGCAGGGATGCAATA |  |  |
| SfaD | CTCCGGAGAACTGGGTGCATCTTAC | 410 |  |
| SfaE | CGGAGGAGTAATTACAAACCTGGCA |  |  |
| fimA_Fw | CGACGCATCTTCCTCATTCTTCT | 721 |  |
| fimA_Rv | ATTGGTTCCGTTATTCAGGGTTGTT |  |  |
| draA_Fw | GCCAACTGACGGACGCAGCAC | 229 |  |
| draA_Rv | CCCCAGCTCCCGACATCGTTTTT |  |  |
| neuB_Fw | CTACCCCTTTTGACGAAGAC | 493 |  |
| neuB_Rv | ACACACCTGACCCCAATAC |  |  |
| kfiC_Fw | GCCACCAACTGTCGCAAAA | 809 |  |
| kfiC_Rv | TGTCGCCCAAACAAAAAGATT |  |  |
| iutA_Fw | GGCTGGACATCATGGGAACTGG | 301 |  |
| iutA_Rv | CGTCGGGAACGGGTAGAATCG |  |  |
| hlyA_Fw | AACAAGGATAAGCACTGTTCTGGCT | 1177 |  |
| hlyA_Rv | ACCATATAAGCGGTCATTCCCGTCA |  |  |
| CDT-up1 | GAAAGTAAATGGAATATAAATGTCCG | 466 | [[3](#_ENREF_3)] [[4](#_ENREF_4)] |
| CDT-up2 | GAAAATAAATGGAACACACATGTCCG |  |  |
| CDT-lp1 | AAATCACCAAGAATCATCCAGTTA |  |  |
| CDT-lp2 | AAATCTCCTGCAATCATCCAGTTA |  |  |
| afa1 | GCTGGGCAGCAAACTGATAACTCTC | 750 | [[5](#_ENREF_5)] |
| afa2 | CATCAAGCTGTTTGTTCGTCCGCCG |  |  |
| cnf-Fw | TTATATAGTCGTCAAGATGGA | 633 | [[4](#_ENREF_4)] |
| cnf-Rv | CACTAAGCTTTACAATATTGA |  |  |
| pks FW | CGCTTCATCAACACGCTTTA | 299 | [[6](#_ENREF_6)] |
| pks Rv | CCATCGCCTATCACCTCAAC |  |  |
| VT1-A (*stx1*) | CGCTGAATGTCATTCGCTCTGC | 302 | [[7](#_ENREF_7)] |
| VT1-B (*stx1*) | CGTGGTATAGCTACTGTCACC |  |  |
| VT2-A (*stx2*) | CTTCGGTATCCTATTCCCGG | 516 |  |
| VT2-B (*stx2*) | CTGCTGTGACAGTGACAAAACGC |  |  |
| EAE-1 (*eae*) | GGAACGGCAGAGGTTAATCTGCAG | 346 |  |
| EAE-2 (*eae*) | GGCGCTCATCATAGTCTTTC |  |  |
| EI1 (*ipaH*) | GCTGGAAAAACTCAGTGCCT | 424 |  |
| EI2 (*ipaH*) | CCAGTCCGTAAATTCATTCT |  |  |
| pCVD432/start (*aggR*) | CTGGCGAAAGACTGTATCAT | 630 |  |
| pCVD432/stop (*aggR*) | CAATGTATAGAAATCCGCTGTT |  |  |
| LT-A-1 (*eltA*) | GGCGACAGATTATACCGTGC | 696 |  |
| LT-A-2 (*eltA*) | CCGAATTCTGTTATATATGTC |  |  |
| STA-1 (*est*) | ATTTTTATTTCTGTATTGTCTTT | 176 |  |
| STA-2 (*est*) | GGATTACAACACAGTTCACAGCAGT |  |  |

**Supplementary Table 3.** Isolated Enterobacteriaceae from tissue samples of ulcerative colitis (UC) and colorectal cancer (CRC) patients, putative invasive strains and confirmed AIEC-like isolates.

| **Patient ID** | **Biopsy location** | **Tissue affectation** | **Isolated Enterobacteriaceae** | **MUG+ indole+** | **MUG- indole+** | **MUG+ indole-** | **MUG- indole-** | **Putative invasive¥** | **Selected for confirmation** | **Confirmed invasive** | **Confirmed AIEC-like** |
| --- | --- | --- | --- | --- | --- | --- | --- | --- | --- | --- | --- |
| **UC patients** |  |  |  |  |  |  |  |  |  |  |  |
| 76 | colon | affected | 45 | 44 | 1 | 0 | 0 | 0 | 0 | 0 | 0 |
| 107 | colon | affected | 48 | 48 | 0 | 0 | 0 | 2 | 2 | 1 | 1 |
| 121 | colon | affected | 91 | 0 | 91 | 0 | 0 | 4 | 4 | 1 | 1 |
| 131 | ileum | unaffected | 80 | 75 | 0 | 5 | 0 | 0 | 2 | 0 | 0 |
|  | colon | affected | 78 | 78 | 0 | 0 | 0 | 4 | 4 | 0 | 0 |
| 157 | ileum | unaffected | 49 | 49 | 0 | 0 | 0 | 0 | 0 | 0 | 0 |
| HSC003 | ileum | unaffected | 85 | 68 | 0 | 0 | 17 | 18 | 10 | 2 | 2 |
|  | colon | affected | 82 | 67 | 0 | 0 | 15 | 16 | 15 | 3 | 3 |
|  | colon | unaffected | 75 | 56 | 1 | 0 | 18 | 12 | 8 | 1 | 1 |
| HSC004 | colon | affected | 87 | 87 | 0 | 0 | 0 | 0 | 0 | 0 | 0 |
| HSC009 | ileum | unaffected | 84 | 84 | 0 | 0 | 0 | 36 | 9 | 1 | 1 |
| HSC015* | colon | affected | 37 | 13 | 2 | 0 | 22 | 0 | 0 | 0 | 0 |
|  | colon | unaffected | 91 | 68 | 2 | 0 | 21 | 0 | 0 | 0 | 0 |
| HSC010 | ileum | unaffected | 91 | 91 | 0 | 0 | 0 | 0 | 0 | 0 | 0 |
|  | colon | affected | 91 | 91 | 0 | 0 | 0 | 0 | 0 | 0 | 0 |
|  | colon | unaffected | 91 | 91 | 0 | 0 | 0 | 0 | 0 | 0 | 0 |
| HSC011 | colon | affected | 79 | 79 | 0 | 0 | 0 | 9 | 9 | 0 | 0 |
|  | colon | unaffected | 91 | 91 | 0 | 0 | 0 | 10 | 10 | 0 | 0 |
| HSC014 | colon | affected | 15 | 0 | 15 | 0 | 0 | 0 | 0 | 0 | 0 |
|  | colon | unaffected | 20 | 0 | 20 | 0 | 0 | 0 | 0 | 0 | 0 |
| HSC013 | ileum | unaffected | 91 | 79 | 5 | 0 | 7 | 15 | 12 | 1 | 0¤ |
|  | colon | affected | 90 | 86 | 0 | 0 | 4 | 6 | 6 | 0 | 0 |
|  | colon | unaffected | 91 | 87 | 0 | 0 | 4 | 15 | 11 | 1 | 0¤ |
| HT002 | colon | affected | 24 | 24 | 0 | 0 | 0 | 13 | 13 | 0 | 0 |
|  | colon | unaffected | 15 | 15 | 0 | 0 | 0 | 0 | 0 | 0 | 0 |
| HT003 | colon | affected | 9 | 1 | 8 | 0 | 0 | 1 | 1 | 0 | 0 |
|  | colon | unaffected | 52 | 24 | 25 | 0 | 3 | 22 | 9 | 5 | 5 |
| **CRC patients** | |  |  |  |  |  |  |  |  |  |  |
| 74 | colon | affected | 96 | 96 | 0 | 0 | 0 | 3 | 6 | 0 | 0 |
| HSC006 | colon | affected | 91 | 91 | 0 | 0 | 0 | 3 | 3 | 0 | 0 |
|  | colon | unaffected | 91 | 80 | 3 | 0 | 8 | 9 | 15 | 0 | 0 |
| HSC007 | colon§ | affected | 91 | 84 | 6 | 0 | 1 | 6 | 5 | 0 | 0 |
|  | colon§ | unaffected | 81 | 81 | 0 | 0 | 0 | 0 | 3 | 0 | 0 |
|  | colon^ | affected | 91 | 88 | 3 | 0 | 0 | 8 | 7 | 0 | 0 |
| HSC012 | colon | affected | 91 | 91 | 0 | 0 | 0 | 0 | 0 | 0 | 0 |
|  | colon | unaffected | 91 | 91 | 0 | 0 | 0 | 0 | 0 | 0 | 0 |
| HSC016 | colon | affected | 91 | 91 | 0 | 0 | 0 | nd | na | na | na |
|  | colon | unaffected | 90 | 90 | 0 | 0 | 0 | nd | na | na | na |
| HSC017 | colon | affected | 81 | 75 | 0 | 6 | 0 | 0 | 0 | 0 | 0 |
|  | colon | unaffected | 77 | 77 | 0 | 0 | 0 | 0 | 0 | 0 | 0 |
| HSC018 | colon | affected | 81 | 81 | 0 | 0 | 0 | 0 | 0 | 0 | 0 |
|  | colon | unaffected | 91 | 91 | 0 | 0 | 0 | 2 | 2 | 0 | 0 |
| HSC019 | colon | affected | 85 | 85 | 0 | 0 | 0 | 0 | 0 | 0 | 0 |
|  | colon | unaffected | 90 | 90 | 0 | 0 | 0 | 14 | 12 | 0 | 0 |
| HSC020 | colon | affected | 91 | 90 | 0 | 1 | 0 | 15 | 10 | 0 | 0 |
|  | colon | unaffected | 91 | 89 | 0 | 2 | 0 | 11 | 11 | 0 | 0 |
| HT001 | colon | affected | 91 | 91 | 0 | 0 | 0 | 14 | 10 | 0 | 0 |
|  | colon | unaffected | 91 | 91 | 0 | 0 | 0 | 1 | 1 | 0 | 0 |
| HSC021 | colon | affected | 91 | 91 | 0 | 0 | 0 | 8 | 8 | 1 | 1 |
|  | colon | unaffected | 86 | 86 | 0 | 0 | 0 | 24 | 10 | 1 | 1 |
| HSC023 | colon | affected | 79 | 14 | 16 | 0 | 49 | 20 | 10 | 0 | 0 |
|  | colon | unaffected | 53 | 0 | 3 | 0 | 50 | 8 | 4 | 0 | 0 |
| HT004 | colon | affected | 91 | 41 | 0 | 0 | 50 | 1 | 1 | 0 | 0 |
|  | colon | unaffected | 69 | 30 | 0 | 0 | 39 | 1 | 1 | 0 | 0 |
| HT005 | colon | affected | 91 | 91 | 0 | 0 | 0 | 68 | 10 | 0 | 0 |
|  | colon | unaffected | 70 | 70 | 0 | 0 | 0 | 11 | 9 | 0 | 0 |
| HT006 | colon | affected | 32 | 32 | 0 | 0 | 0 | 5 | 3 | 0 | 0 |
|  | colon | unaffected | 16 | 16 | 0 | 0 | 0 | 3 | 3 | 0 | 0 |
| ¥ strains of category 3 determined by the qualitative invasion assay; * HSC015 is patient HSC009 one year after the first colonoscopy; § transversal; ^descendent | | | | | | | | | | | |
| MUG= β-glucuronidase activity; ¤ not confirmed as *E. coli.* | | | |  |  |  |  |  |  |  |  |
| nd: no data, strains were resistant to gentamicin and kanamycin. | | | | |  |  |  |  |  |  |  |
| na: not applicable | |  |  |  |  |  |  |  |  |  |  |

**Supplementary Table 4.** Adhesion and invasion indices of strains from Coeliac (CeD) and healthy (H) children obtained in a previous work [[8](#_ENREF_8)], assayed on Intestine-407 cells.

| **ISOLATE** | **Id Patient** | **ADH_I (mean)** | **ADH_I (SD)** | **INV_I (mean)** | **INV_I (SD)** | **REPL_I (mean)** | **REPL_I (SD)** | **Phenotype** | **Phylogroup** | ***fimA*** | ***sfaD/E*** | ***papC*** | ***draA*** | ***hlyA*** | ***kfiC*** | ***neuB*** | ***iutA*** |
| --- | --- | --- | --- | --- | --- | --- | --- | --- | --- | --- | --- | --- | --- | --- | --- | --- | --- |
| **CeD Active** |  |  |  |  |  |  |  |  |  |  |  |  |  |  |  |  |  |
| ENT CAI 1 | CAI | 9.17 | 1.74 | 0.050 | 0.031 | nd |  | Non-AIEC | B2 | + | - | - | - | - | - | - | - |
| ENT CAI 5 | CAI | 14.67 | 4.54 | 0.030 | 0.027 | nd |  | Non-AIEC | B2 | + | - | - | - | - | + | - | - |
| ENT CAK 1 | CAK | 0.88 | 0.49 | 0.018 | 0.006 | nd |  | Non-AIEC | A | + | - | - | - | - | - | - | - |
| ENT CAK 3 | CAL | 0.92 | 0.55 | 0.023 | 0.010 | nd |  | Non-AIEC | A | + | - | - | - | - | - | - | + |
| ENT CAL 2 | CAL | 2.87 | 0.12 | 0.070 | 0.027 | nd |  | Non-AIEC | A | + | - | - | - | - | - | - | - |
| ENT CAL 5 | CAL | 0.09 | 0.06 | 0.001 | 0.000 | nd |  | Non-AIEC | B2 | + | + | - | - | - | - | + | + |
| ENT CAP 1 | CAP | 0.52 | 0.28 | 0.012 | 0.007 | nd |  | Non-AIEC | B2 | + | - | + | - | - | - | + | - |
| ENT CBD 1 | CBD | 0.24 | 0.14 | 0.025 | 0.018 | nd |  | Non-AIEC | B2 | + | - | - | - | - | - | - | - |
| ENT CBD 10 | CBD | 0.38 | 0.29 | 0.022 | 0.011 | nd |  | Non-AIEC | A | - | - | - | - | - | - | - | - |
| ENT CBE 10 | CBE | 0.17 | 0.11 | 0.004 | 0.000 | nd |  | Non-AIEC | D | + | - | - | - | - | - | - | + |
| ENT CBE 9 | CBE | 0.96 | 0.62 | 0.071 | 0.074 | nd |  | Non-AIEC | B2 | + | - | - | - | - | - | - | - |
| ENT CBG 3 | CBG | 0.09 | 0.01 | 0.038 | 0.027 | nd |  | Non-AIEC | A | - | - | - | - | - | - | - | - |
| ENT CCI 1 | CCI | 0.77 | 0.04 | 0.026 | 0.000 | nd |  | Non-AIEC | D | - | + | - | - | + | + | - | - |
| ENT CCM 1 | CCM | 1.73 | 0.58 | 0.053 | 0.028 | nd |  | Non-AIEC | A | + | - | - | - | - | - | - | - |
| **CeD Inactive** |  |  |  |  |  |  |  |  |  |  |  |  |  |  |  |  |  |
| ENT CBJ 1 | CBJ | 2.10 | 0.63 | 0.008 | 0.006 | nd |  | Non-AIEC | A | - | - | - | - | - | - | - | - |
| ENT CBL 2 | CBL | 0.95 | 0.35 | 0.002 | 0.000 | nd |  | Non-AIEC | D | + | + | + | - | + | - | - | - |
| ENT CBM 1 | CBM | 1.39 | 0.51 | 0.040 | 0.019 | nd |  | Non-AIEC | D | + | + | + | - | + | - | + | - |
| ENT CBN 1 | CBN | 0.40 | 0.10 | 0.020 | 0.013 | nd |  | Non-AIEC | B2 | + | - | - | - | - | - | - | - |
| ENT CBN 8 | CBN | 0.46 | 0.07 | 0.012 | 0.005 | nd |  | Non-AIEC | B2 | - | - | - | - | - | - | - | - |
| ENT CCC 1 (+4) | CCC | 0.12 | 0.05 | 0.007 | 0.005 | nd |  | Non-AIEC | D | - | - | - | - | - | - | - | - |
| ENT CCC 4 | CCC | 0.13 | 0.02 | 0.007 | 0.005 | nd |  | Non-AIEC | D | + | - | - | - | - | - | - | - |
| ENT CCD 1 (+2) | CCD | 2.53 | 1.01 | 0.021 | 0.019 | nd |  | Non-AIEC | A | + | + | - | - | - | - | + | - |
| ENT CCH 1 | CCH | 0.30 | 0.16 | 0.002 | 0.002 | nd |  | Non-AIEC | D | + | - | - | - | - | - | - | + |
| ENT CCH 3 | CCH | 0.39 | 0.16 | 0.003 | 0.002 | nd |  | Non-AIEC | D | - | - | - | - | - | - | - | - |
| ENT CCJ 1 | CCJ | 0.09 | 0.05 | 0.000 | 0.000 | nd |  | Non-AIEC | A | + | - | - | - | - | - | - | - |
| ENT CCJ 5 | CCJ | 0.15 | 0.11 | 0.001 | 0.001 | nd |  | Non-AIEC | A | + | + | - | - | + | - | - | + |
| **Healthy children** | |  |  |  |  |  |  |  |  |  |  |  |  |  |  |  |  |
| ENT CAJ 1 | CAJ | 3.63 | 1.69 | 4.800 | 0.950 | 882.8 | 180.5 | AIEC | B2 | + | - | - | - | - | - | - | + |
| ENT SAB 5 | SAB | 0.08 | 0.06 | 0.023 | 0.019 | nd |  | Non-AIEC | A | + | - | - | - | - | - | - | - |
| ENT SAD 1 (4) | SAD | 0.07 | 0.02 | 0.016 | 0.012 | nd |  | Non-AIEC | B2 | + | - | - | - | - | - | - | - |
| ENT SAF 3 | SAF | 1.27 | 1.04 | 1.150 | 1.021 | 399.4 | 41.3 | AIEC | D | - | - | - | - | - | - | - | - |
| ENT SAG 5 | SAG | 0.04 | 0.04 | 0.001 | 0.001 | nd |  | Non-AIEC | A | - | - | - | - | - | - | - | - |
| ENT SAH 4 | SAH | 0.92 | 0.62 | 0.007 | 0.003 | nd |  | Non-AIEC | D | - | - | - | - | - | - | - | - |
| ENT SR 1 | SR | 0.12 | 0.17 | 0.002 | 0.001 | nd |  | Non-AIEC | B2 | + | + | - | - | + | - | - | - |
| ENT SR 2 | SR | 0.33 | 0.14 | 0.014 | 0.003 | nd |  | Non-AIEC | B2 | + | + | - | - | - | - | + | - |
| ENT SS 1 | SS | 0.52 | 0.38 | 0.010 | 0.006 | nd |  | Non-AIEC | D | - | + | - | - | - | - | - | - |
| ENT SS 2 | SS | 1.05 | 0.35 | 0.006 | 0.004 | nd |  | Non-AIEC | D | - | + | - | - | - | + | + | - |
| ENT ST 1 | ST | 0.10 | 0.09 | 0.005 | 0.003 | nd |  | Non-AIEC | B1 | + | - | + | - | + | - | - | + |
| ENT ST 5 | ST | 0.03 | 0.02 | 0.001 | 0.001 | nd |  | Non-AIEC | A | - | - | - | - | - | - | - | - |

# Supplementary Figures

| 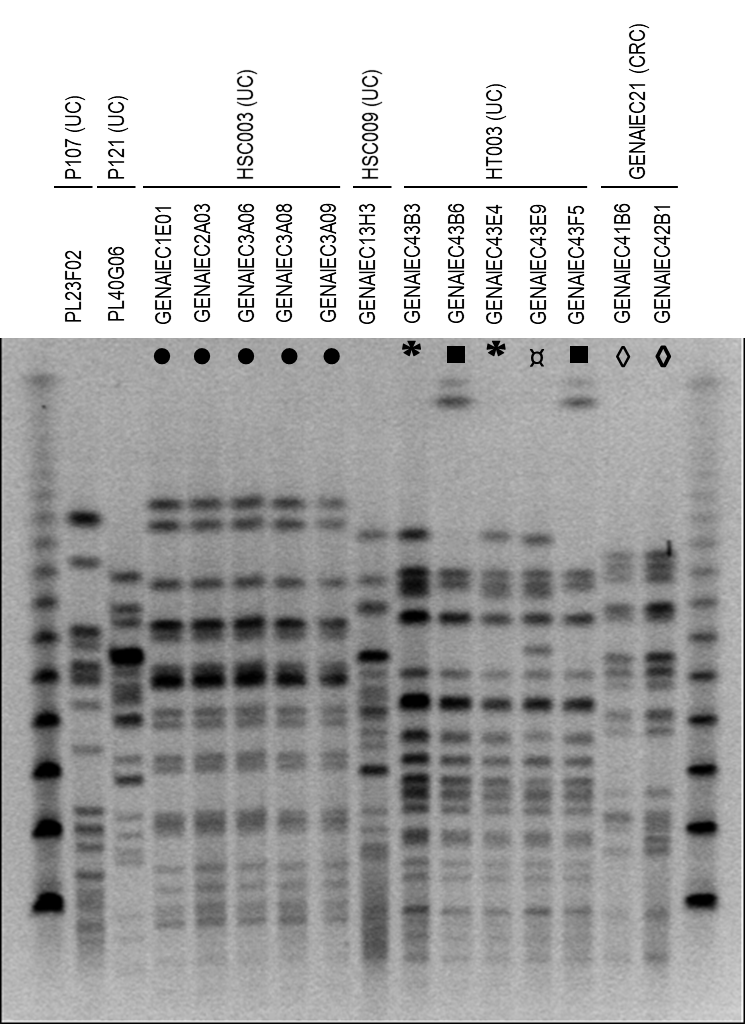 |
| --- |
| **Supplementary Figure 1.** Pulsed Field Gel Electrophoresis (PFGE) profiles of AIEC-like isolates obtained from UC and CRC patients. Symbols represent isolates with identical PFGE. Only for 15/16 adherent-invasive isolates confirmed to be *E. coli* were analyzed. |

**References of the supplementary materials**

1. Clermont, O., S. Bonacorsi, and E. Bingen, *Rapid and Simple Determination of the Escherichia coli Phylogenetic Group.* Appl Environ Microbiol, 2000. **66**(10): p. 4555-4558.

2. Nowrouzian, F., I. Adlerberth, and A.E. Wold, *P fimbriae, capsule and aerobactin characterize colonic resident Escherichia coli.* Epidemiol Infect, 2001. **126**(1): p. 11-18.

3. Allué-Guardia, A., L. Imamovic, and M. Muniesa, *Evolution of a self-inducible cytolethal distending toxin type V-encoding bacteriophage from Escherichia coli O157:H7 to Shigella sonnei.* Journal of virology, 2013. **87**(24): p. 13665-13675.

4. Tóth, I., et al., *Production of Cytolethal Distending Toxins by Pathogenic Escherichia coli Strains Isolated from Human and Animal Sources: Establishment of the Existence of a New cdt Variant (Type IV).* Journal of Clinical Microbiology, 2003. **41**(9): p. 4285-4291.

5. Mora, A., et al., *Poultry as reservoir for extraintestinal pathogenic Escherichia coli O45:K1:H7-B2-ST95 in humans.* Veterinary Microbiology, 2013. **167**(3): p. 506-512.

6. Ewers, C., et al., *Intestine and Environment of the Chicken as Reservoirs for Extraintestinal Pathogenic Escherichia coli Strains with Zoonotic Potential.* Appl Environ Microbiol, 2009. **75**(1): p. 184-192.

7. Blanco, M., et al., *Identification of two new intimin types in atypical enteropathogenic Escherichia coli.* Int Microbiol, 2006. **9**(2): p. 103-110.

8. Sanchez, E., et al., *Reduced diversity and increased virulence-gene carriage in intestinal enterobacteria of coeliac children.* BMC Gastroenterol, 2008. **8**(1): p. 50.
